# Progress of Biology in England

**Published:** 1881-11

**Authors:** John Lubbock


### PROGRESS OF BIOLOGY IN ENGLAND.

The Biological Section is that with which I have been most intimately associated, and with which it is, perhaps, natural that I should begin. Fifty years ago it was the general opinion that animals and plants came into existence just as we now see them. We took pleasure in their beauty; their adaptation to their habits and mode of life in many cases could not be overlooked or misunderstood. Nevertheless, the book of Nature was like some richly illuminated missal, written in an unknown tongue; the graceful forms of the letters, the beauty of the coloring, excited our wonder and admiration; but of the true meaning little was known to us; indeed, we scarcely realized that there was any meaning to decipher. Now glimpses of the truth are gradually revealing themselves; we perceive that there is a reason—and in many cases we know what that reason is—for every difference in form, in size, and in color; for every bone and every feather, almost for every hair. Moreover, each problem which is solved opens out vistas, as it were, of others perhaps even more interesting. With this great change the name of our illustrious countryman, Darwin, is intimately associated, and the year 1859 will always be memorable in science as having produced his great work on "The Origin of Species." In the previous year he and Wallace had published short papers, in which they clearly state the theory of natural selection, at which they had simultaneously and independently arrived. We can not wonder that Darwin's views should have at first excited great opposition. Nevertheless, from the first they met with powerful support, especially in this country, from Hooker, Huxley, and Herbert Spencer. The theory is based on four axioms:

"1. That no two animals or plants in nature are identical in all respects. 2. That the offspring tend to inherit the peculiarities of their parents. 3. That of those which come into existence, only a small number reach maturity. 4. That those which are, on the whole, best adapted to the circumstances in which they are placed, are most likely to leave descendants."

Darwin commenced his work by discussing the causes and extent of variability in animals, and the origin of domestic varieties; he showed the impossibility of distinguishing between varieties and species, and pointed out the wide differences which man has produced in some cases—as, for instance, in our domestic pigeons, all unquestionably descended from a common stock. He dwelt on the struggle for existence (which has since become a household word), and which, inevitably resulting in the survival of the fittest, tends gradually to adapt any race of animals to the conditions in which it occurs. While thus, however, showing the great importance of natural selection, he attributed to it no exclusive influence, but fully admitted that other causes — the use and disuse of organs, sexual selection, etc—had to be taken into consideration.—Sir JOHN LUBBOCK, *in Popular Science Monthly for November.*

